# Predictive value of less than moderate residual mitral regurgitation as assessed by transesophageal echocardiography for the short-term outcomes of patients with mitral regurgitation treated with mitral valve repair

**DOI:** 10.1186/1476-7120-5-25

**Published:** 2007-07-20

**Authors:** Antonio Rizza, Laureta Sulcaj, Mattia Glauber, Giuseppe Trianni, Cataldo Palmieri, Massimiliano Mariani, Stefano Maffei, Sergio Berti

**Affiliations:** 1 CNR-Institute of Clinical Physiology, G. Pasquinucci Hospital, Massa, Italy

## Abstract

**Background:**

Traditionally, in patients with mitral regurgitation (MR) a successful mitral valve repair is considered when residual MR by post-pump transesophageal echocardiography (TEE) is less than moderate or absent. Little is known about the prognostic value of less than moderate (mild or mild-to-moderate) residual MR for the early outcome of patients treated with mitral valve repair.

**Methods:**

Eligible for this study were patients undergoing isolated mitral valve repair. Patients with moderate or severe residual MR after valve repair were excluded. The primary endpoint of the study was the composite of death or need of reintervention.

**Results:**

A total of 98 patients (54 with no residual MR-Group 1, and 44 with less than moderate residual MR-Group 2) were analyzed. Of these, 72% presented with New York Heart Association (NYHA) 3/4, and 38% were women. The primary endpoint of the study occurred in 3 (5.5%) patients in Group 1 and 6 (13.6%) patients in Group 2 MR (*P *= 0.31). There was a trend toward a higher incidence of use of inotropic drugs post-interventional (*P *= 0.12), and a longer hospital stay among patients with less than moderate residual MR (*P *= 0.18).

**Conclusion:**

In our study population, patients with less than moderate residual MR had a trend toward a higher risk of early adverse outcomes as compared with patients with no residual MR by post-pump TEE. Studies with a larger patient population and longer follow-up data may be useful to better define the clinical significance of residual mild MR after mitral vale repair.

## Background

Mitral regurgitation is the most commonly encountered valve lesion in modern clinical practice [1]. As echocardiography is the most widely available cardiac imaging modality, it is the technique which is routinely used to assess patients with suspected or known MR. Echo-Doppler is an excellent technique for detecting the presence of MR and defining the underlying pathological cause [2]. Echo-Doppler grading of regurgitation severity in conjunction with patient symptoms and signs and occasionally invasive haemodynamic information are useful in the decision-making process with respect to the need for and timing of mitral valve surgery [2].

It is important to note that mitral regurgitation changed in the last decades with regard to its etiologic profile, which is now dominated by degenerative and ischemic causes in developed countries [3]; to its noninvasive assessment with the developments in transesophageal echocardiography (TEE), colour flow imaging, and new methods of quantization of regurgitation [1]; and to its management with improved understanding of the role of left ventricle (LV) function in prognosis [2]. Most importantly, advances in conservative surgery have improved its treatment [4].

The post-Cardiac Pulmonary Bypass (CPB) TEE exam is essential in helping to determine the competency of the repaired mitral valve (MV), and to evaluate persistent MR [5]. Hemodynamic loading conditions and left ventricle function also must be taken into consideration in the assessment of residual MR [6]. Following MV procedures, several studies have suggested that, in approximately 5–11% of cases the post-CPB TEE exam may identify persistent lesions that require additional immediate surgical intervention [7,8]. Moderate to severe residual MR will necessitate a return to CPB for further evaluation and repeat surgery.

The aim of the present retrospective study was to assess the short-term outcome of patients who underwent mitral valve repair in our Cardiac Surgery Division with less than moderate mitral regurgitation after repair.

## Methods

### Data source

To collect the necessary information for this study we used the database of patients admitted and treated at Ospedale Pasquinucci, IFC-CNR, Massa, Italy from January 2004 to October 2005.

### Patient population

Eligible for inclusion in this study were considered all patients who consecutively underwent mitral valve replacement for mitral regurgitation with no or less than moderate residual post-pump mitral regurgitation at the service of cardiac surgery for the adults at our Institution. Patients who underwent mitral valve repair in conjunction with other planned cardiovascular surgical interventions such as coronary bypass artery grafting, other cardiac valve surgery, previous mitral valve repair, interventions for abdominal or thoracic aortic disease or accompanying congenital malformations were excluded from the study. On the other hand, no restriction criteria were imposed with respect to age, symptoms at baseline or presence of concomitant diseases. Although this was a retrospective study, all data have been prospectively collected and entered into the above-mentioned hospital database. Overall, 98 patients with no or mild residual mitral regurgitation were included in this study. These were divided into two groups: those with no residual mitral regurgitation and those with less than moderate (mild and mild-to-moderate) residual mitral regurgitation. The first group consisted of 54 patients and the second group consisted of 44 patients.

### Etiology of mitral regurgitation

Possible causes included primary valve and functional causes: mitral valve prolapse, rheumatic heart disease, endocarditis, papillary muscle rupture, congenital, mitral annular dilatation, left ventricular wall motion abnormality. Patients with previous mitral valve repair were excluded from the study.

### Methodology used for the measurement of mitral regurgitation

The severity of mitral regurgitation was graded according the recommendation of the American Society of Echocardiography [9]. Preoperative transthoracic assessment was performed using a General Electrics Vivid 7 dimension echographer with a 3 MHz cardiologic probe (General Electrics mod. 2323337 3S). Postoperative assessment was performed at the end of cardiopulmonary by-pass with a Philips Sonos 7500 echographer and a 5 MHz transoesophageal multiplane transducer (Hewlett Packard mod.21378A). Postoperative mitral regurgitation was evaluated as stated in guidelines [9] with a semiquantitative grading from absent to severe.

### Endpoints and definitions

The primary endpoint of the study was the composite of death and need of reintervention for recurrent severe mitral regurgitation. Other outcomes of interest were the need for intraoartic balloon pump and inotropic drugs in the immediate period after the intervention, intubation time (time to extubation after the intervention) and length of stay in the intensive care unit as well as total length of hospital stay. Other complications such as bleeding, ischemic complications, respiratory complications, renal failure and wound infection were also evaluated. The type of required reintervention was also documented. Arterial hypertension was defined as systolic blood pressure of ≥140 mmHg and/or diastolic blood pressure of ≥90 mmHg on at least 2 separate occasions or the treatment with antihypertensive agents. Hypercholesterolemia was defined as total cholesterol ≥240 mg/dl or treatment with a lipid-lowering agent.

### Statistical analysis

All data presented are shown as mean ± standard deviation or numbers (percentages). To compare the differences between groups, we used student *t *test for continuous data and chi-square or Fisher's exact test for categorical data, as appropriate. Statistical significance was accepted for a two-tailed P < 0.05.

## Results

### Baseline clinical characteristics

A total of 98 patients, 54 patients with no residual mitral regurgitation and 44 patients with less than moderate residual mitral regurgitation of were available for the analysis. Most of the patients presented with NYHA 3/4 (62%). Women constituted 38% of the study population while 27% of the patients had diabetes mellitus. Around 41% of the patients included in this study had hyperlipidemia, 49% had arterial hypertension while 17% of them were current smokers. Mean body mass index, was 29 ± 3.2 kg/m^2 ^for this patient population. From the overall study population, 12% of the patients had angiographically documented coronary artery disease and 11% of them had a history of prior myocardial infarction. From the total study population, 13% of the patients had previously undergone a cardiovascular intervention (coronary bypass artery grafting, percutaneous coronary interventions or surgical treatment for aortic disease). Atrial fibrillation was diagnosed in 25% of the patients at baseline. There were no statistically significant differences with respect the baseline clinical and characteristics between the patients who had no residual mitral regurgitation or less than moderate residual mitral regurgitation at the post-pump TEE (Table 1).

**Table 1:** Baseline characteristics.

**Variables**	**Group 1 (n = 54)**	**Group 2 (n = 44)**	***P value***
Age, years	62.3 ± 11.5	66.6 ± 10.5	0.11
Women, n (%)	19 (35)	18 (41)	0.56
Weight, kg	73.4 ± 14.7	68.9 ± 13.7	0.13
Height, cm	172.1 ± 9.0	168.9 ± 8.9	0.07
Body mass index, kg/m^2^	24.9 ± 3.9	24.0 ± 3.4	0.40
Current smoker, n (%)	10 (19)	7 (16)	0.73
Diabetes mellitus, n (%)	15 (28)	11 (25)	0.76
Hyperlipidemia, n (%)	22 (41)	17(39)	0.83
Arterial hypertension, n (%)	22 (41)	24 (55)	0.17
Ejection fraction, %	57.9 ± 9.4	55.3 ± 8.3	0.13
NYHA 3/4, n (%)	30 (56)	31 (70)	0.20
COPD, n (%)	9 (17)	6 (14)	0.68
Other concomitant diseases, n (%)	13 (19)	10 (16)	0.88

NYHA-New York Heart Association (classification). COPD-chronic obstructive pulmonary disease. Group 1-no residual mitral regurgitation. Group 2-less than moderate mitral regurgitation.

### Baseline echocardiographic characteristics (transthoracic echocardiogram)

Of the total patient population, 50% had moderate MR and 50% severe MR. Mean pulmonary artery systolic pressure was 41 ± 8.9 mmHg and mean left atrium size 45 ± 6.3 mm. Mean ejection fraction of left ventricle was 57% in the total study population. Similar to the baseline clinical characteristics, there was no statistically significant difference between the 2 study groups with respect to baseline transthoracic echocardiographic indexes (Table 2).

**Table 2:** Baseline echocardiographic data (transthoracic ECHO)

**Variables**	**Group 1 (n = 54)**	**Group 2 (n = 44)**	***P value***
Mitral regurgitation			0.68
Moderate, n (%)	26 (48)	23 (52)	
Severe, n (%)	28 (52)	21 (48)	
PASP, mmHg	40.9 ± 8.8	41.6 ± 10.6	0.70
Left atrium size, mm	44.9 ± 6.4	45.8 ± 6.1	0.49

PSAP-pulmonary artery systolic pressure.

### Causes of mitral regurgitation (transesophageal echocardiography)

In most of the cases the cause of MR was mitral valve prolapse (73%). The second most frequent cause was left ventricular wall motion abnormalities, which were present in 25% of the patients. Mitral annular dilatation (11%), papillary muscle rupture (5%), rheumatic heart disease (4%), endocarditis (4%) and congenital anomaly of mitral valve (1%) were less frequently reported. In some patients more than one of the above-mentioned causes of MR was present. These causes were non-differently distributed between patients with no residual MR and patients less than moderate residual MR (Table 3).

**Table 3:** Causes of mitral regurgitation (transesophageal ECHO).

**Causes**	**Group 1 (n = 54)**	**Group 2 (n = 44)**	***P value***
Mitral valve prolapse, n (%)	43 (80)	29 (66)	0.13
Rheumatic heart disease, n (%)	2 (4)	2 (5)	0.82
Endocarditis, n (%)	3 (6)	1 (2)	0.78
Chordal rupture, n (%)	4 (7)	1 (2)	0.50
Congenital anomaly, n (%)	1 (2)	0 (0)	0.91
Mitral annular dilatation, n (%)	6 (11)	5 (11)	0.97
Left ventricular wall motion abnormality, n (%)	13 (24)	11 (25)	0.92

### In hospital outcomes, complications and re-interventions

**Figure 1:**
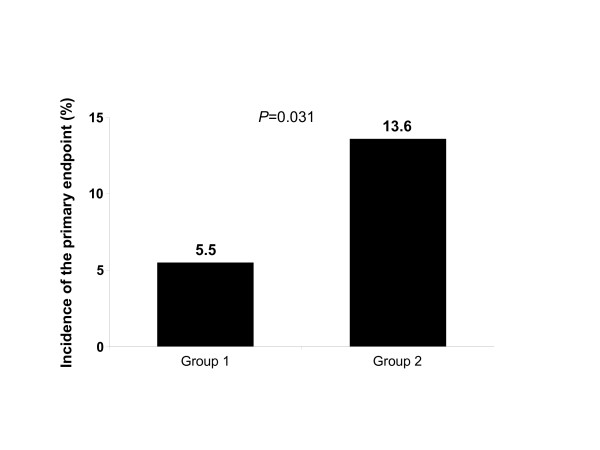
Incidence of the primary endpoint (composite of in-hospital death or need of reintervention) in Group 1 (patients with no residual mitral regurgitation) and Group 2 (less than moderate residual mitral regurgitation).

The primary endpoint of the study, the composite of in-hospital death or need of reintervention for recurrent severe mitral regurgitation in 3 (5.5%) patients with no residual MR and 6 (13.6%) patients with less than moderate residual MR (Figure 1, P = 0.31). In the group with no residual MR 1 (2%) patient died and another patient suffered a stroke. There were no deaths or strokes among patients with less than moderate residual MR, but 1 (2%) patient in this group had a thrombotic episode of an inferior limb which was treated percutaneously. Two (3.7%) patients with no residual MR and 6 (13.6%) of the patients with less than moderate residual MR had recurrent severe mitral regurgitation (P = 0.16). Patients with no residual MR underwent mitral valve replacement while 3 of the 6 patients with less than moderate residual MR underwent repeat valve replacement and the other 3 patients underwent mitral valve repair. Bleeding complications were reported in 2 (4%) patients in the first group and 2 (%) in the second group (P = 0.85). These patients were either re-opened or had a drain placed. Any of the following complications: recurrent several mitral regurgitation, bleeding, cognitive damage, respiratory complications, stroke limb ischemia, wound infection or renal failure occurred in 9 (17%) patients with no residual MR and 15 (34%) patients with less than moderate residual MR (P = 0.08) (Tables 4, 5 and 6).

**Table 4:** In hospital outcomes.

**Outcomes**	**Group 1 (n = 54)**	**Group 2 (n = 44)**	***P value***
Death, n (%)	1 (2)	0 (0)	0.91
Intraaortic balloon, n (%)	2 (4)	2 (5)	0.85
Inotropic drugs, n (%)	21 (39)	55 (47)	0.12
Intubation time, hours	9.3 ± 6.9	12.6 ± 9.2	0.04
Length of stay in ICU, days	1.3 ± 1.3	1.4 ± 0.7	0.84
Total hospital stay, days	8.7 ± 3.3	9.7 ± 3.8	0.18

ICU-intensive care unit.

**Table 5:** Complications.

**Complications**	**Group 1 (n = 54)**	**Group 2 (N = 44)**	***P value***
Bleeding, n (%)	2 (4)	2 (5)	0.85
Recurrent severe mitral regurgitation, n (%)	2 (4)	6 (14)	0.16
Cognitive damage, n (%)	2 (4)	3 (7)	0.81
Respiratory complications, n (%)	2 (4)	4 (9)	0.49
Stroke, n (%)	1 (2)	0 (0)	0.91
Wound infection, n (%)	1 (2)	3 (7)	0.47
Limb ischemia, n (%)	0 (0)	1 (2)	0.90
Renal insufficiency, n (%)	0 (0)	1 (2)	0.90
Any of the above, n (%)	9 (17)	15 (34)	0.08
Other, %	2 (4)	2 (5)	0.85

ICU-intensive care unit. *Other *includes, laceration of aorta during the intervention and subcutaneous emphysema.

**Table 6:** Reinterventions.

**Reinterventions**	**Group 1 (n = 54)**	**Group 2 (N = 44)**	***P value***
Total reinterventions, n (%)	4 (7)	7 (16)	0.32
Reintervention for bleeding, n (%)	2 (4)	1 (2)	0.87
Mitral valve replacement, n (%)	2 (4)	3 (7)	0.81
Mitral valve repair, n (%)	0	3 (7)	0.17

Only 2 patients in each group required implantation of intraaortic balloon (4% vs 5%, P = 0.85). A marginally non-significant difference was observed with regard to the need for inotropic drugs (P = 0.12). Patients with less than moderate residual MR had a marginally significant longer intubation time (P = 0.04). Mean length of stay in the ICU and total length of stay were not significantly different between the 2 groups (respectively P = 0.84 and P = 0.18, Table 6).

## Discussion

In this study we evaluated the prognostic value for the early adverse outcomes of mild residual MR, as assessed by post-pump transesophageal echocardiography, after valve repair in patients with mitral valve regurgitation. We found an increased risk, though statistically not significant, among patients with less than moderate MR as compared to those with no residual mitral regurgitation with respect to the composite of early adverse outcomes as well as postintervention complications. Our findings suggest that in a patient population with characteristics similar to the one evaluated in our study, less than moderate residual regurgitation might negatively influence the outcome of patients who have undergone mitral vale repair. This hypothesis should be further explored.

The post-CPB TEE examination plays a major role in the establishing the competency of the repaired MV, and to evaluate persistent MR. Hemodynamic loading conditions and LV function must also be taken into consideration in the assessment of residual MR. Following MV procedures, several studies have suggested that, in approximately 5–11% of cases the post-CPB TEE exam may identify persistent lesions that require additional immediate surgical intervention [7,8]. Residual moderate or severe MR will usually necessitate a return to CPB for further evaluation and definitive surgery. However, little is known about the outcome of patients who have less than moderate residual MR after repair evaluated by means of intraoperative TEE.

In an early study, Fix et al. analyzed early outcomes among 76 patients with grade mild-to-moderate residual MR by post-pump intraoperative echocardiography and compared them with 76 well-matched patients who had no residual MR by post-pump echocardiography [10]. In-hospital morbidity measured by the frequency of respiratory complications, strokes, time in intensive care unit, and duration of hospital stay was reported to be higher in the patients with no residual MR after repair. Hospital mortality was not significantly different. Similar to this study we found no difference in in-hospital mortality among patients with different grades of residual MR. However, differently from this study we did not found a significant difference in in-hospital morbidity between patients with no residual MI and less than moderate residual MI. Moreover, we found a trend favouring patients with no residual MI with respect to the need of reintervention for recurrent severe mitral regurgitation and a composite of postintervention complications. The different results between our study and that of Fix et al. [10] should be sought in the different patients included (e.g. in contrast to Fix et al. we excluded patients who underwent concomitant cardiovascular surgery) as well as different time periods in which patients of the respective studies were operated (more than 15 years difference) which may reflect differences in valve repair techniques, operator skills, pre- and post-operative care and adjunctive treatments.

Only 1 (1%) patient died in the early peri-interventional period in our study. This is consistent with the reports of literature according to which the outcome of mitral valve repair is dependent on the underlying disease with an operative mortality after mitral valve repair ranging from 0–5% in patients with degenerative MR [11] to 7–26% in patients with ischemic MR [12]. In our study, in the majority of the patients that were included the cause of MR was degeneration of MV.

The low risk profile our study population as well the small number of patients that were evaluated, may be responsible for the lack of differences between the 2 groups in our study. As stated above, there was a trend toward a more frequent need for repeat reinterventions for recurrent severe mitral regurgitation among patients with mild residual MI (P = 0.16) with even a more pronounced trend when the composite of complications was assessed (P = 0.08). On the other hand, although both groups were well matched with respect to the baseline clinical and echocardiographic characteristics and causes of MR, there might have been important occult differences between the 2 study groups. A limitation of this study is also the lack of a longer clinical follow-up.

## Conclusion

In a patient population with characteristics similar to that evaluated in our study, less than moderate residual MR by post-pump TEE after mitral valve repair for mitral regurgitation appears to confer an increased risk for adverse outcomes during the early post-intervention period as compared to the lack of residual MR. Studies with a larger patient population and longer follow-up data are required to better characterize the clinical importance of less than moderate residual MR after mitral vale repair.

## Authors' contributions

AR conceived of the study, and participated in its design and coordination. LS participated in design and performed the statistical analysis. MG carried out the mitral valve replacement at cardiac surgery. GT participated in the patient's selection and echocardiographic analysis. CP participated in the patient's selection and echocardiographic analysis. MM participated in the patient's selection and echocardiographic analysis. SM participated in the patient's selection and echocardiographic analysis. SB conceived of the study, and participated in its design and coordination.

All authors read and approved the final manuscript.
